# Neurorehabilitation of Traumatic Brain Injury (TBI): A Clinical Review

**DOI:** 10.3390/medsci7030047

**Published:** 2019-03-18

**Authors:** Michael Oberholzer, René M. Müri

**Affiliations:** 1 Department of Neurology, University Neurorehabilitation, Inselspital Bern, Bern University Hospital and University of Bern, 3010 Bern, Switzerland; rene.mueri@dbmr.unibe.ch; 2 ARTORG Center for Biomedical Engineering Research, University of Bern, 3010 Bern, Switzerland

**Keywords:** traumatic brain injury, neurorehabilitation, disorders of consciousness, paroxysmal sympathetic hyperactivity, posttraumatic agitation, posttraumatic hydrocephalus, posttraumatic neuroendocrine disorders, cognitive rehabilitation, neuropsychological rehabilitation

## Abstract

Traumatic brain injury (TBI) and its potential long-term consequences are of major concern for public health. Neurorehabilitation of affected individuals has some specific characteristics in contrast to neurorehabilitation of patients with acquired brain lesions of other aetiology. This review will deal with the clinical consequences of the distinct lesions of TBI. In severe TBI, clinical course often follows a typical initial sequence of coma; followed by disturbed consciousness; later, post-traumatic agitation and amnesia; and finally, recovery of function occurs. In the different phases of neurorehabilitation, physicians should be aware of typical medical complications such as paroxysmal sympathetic hyperactivity, posttraumatic hydrocephalus, and posttraumatic neuroendocrine dysfunctions. Furthermore, we address questions on timing and on existing evidence for different rehabilitation programmes and for holistic neuropsychological rehabilitation approaches.

## 1. Introduction

Recent studies [1,2] estimated that over 40% of patients who were hospitalized as a result of an acute moderate to severe traumatic brain injury (TBI) show long-term disability, with prevalence rates ranging from 3.2 to 5.3 million in the USA, that is, more than 1.1% of the U.S. population. In Switzerland, estimated incidence of severe TBI (as defined by the Abbreviated Injury Scale of the head, score > 3) was 10.58 per 100,000 inhabitants per year [3]. Data on the economic impact of this problem are scarce, but estimates for the USA rate the annual cost to be more than 221 billion U.S. dollars, while estimates of the Center of Disease Control being more conservative with 56 billion U.S. dollars [4]. The total impact may well be underestimated because of the neglect of indirect costs (care and support services by caregivers and family members). Thus, not only from the point of view of the affected individual, but also from an economic point of view, it seems reasonable to support the recovery of affected individuals and help them to regain their independence by means of an optimized rehabilitation period.

Neurorehabilitation of TBI is a vast, many-sided topic—ranging from early rehabilitation of patients with impaired consciousness to support and to accompany reintegration of patients in their social and professional environments. Reviewing the relevant existing literature addressing the different aspects of rehabilitation of TBI in a balanced way is thus not feasible for a short review article. Therefore, we will focus on in-patient rehabilitation and some specific topics that are clinically relevant in neurorehabilitation of TBI, and point out what distinguishes it from the care of patients suffering from other brain injuries.

## 2. Lesion Diversity and Clinical Patterns

### 2.1. From Lesion Diversity…

One might think that neurorehabilitation of any brain injury, independent from its aetiology (ischemic, haemorrhagic, trauma, or hypoxic), should be similar. However, this is not the case, as the mechanisms of brain damage and localisation of the lesions are quite different between the different groups. Ischemic stroke leads to damage of the brain, which is determined by the vasculature, hypertensive haemorrhagic damage may show some locations of predilection, but may be localized almost anywhere in the brain. By contrast, TBI often results in bi-hemispheric contusions and lesions, some of them very localized (hematomata in the parenchyma) or in the subarachnoid, subdural, or epidural space, respectively. Furthermore, typically, widespread injuries of cell bodies and axons occur, the latter often being referred to as diffuse axonal injury (DAI) [5,6,7,8,9,10]. These types of damage are followed by relevant diffuse œdema of the brain, with multiple adaptations on cellular and metabolic levels [8,11]. 

Standard magnetic resonance imaging (MRI) sequences typically lead to an underestimate of the damage even in the presence of cognitive dysfunction [12]. However, in a Norwegian study that included 106 patients receiving imaging within four weeks post TBI, the severity of DAI showed some correlation with lower Glasgow outcome scale-extended (GOSE) score one year post-injury [13].

### 2.2. … To Clinical Patterns

On the one hand, focal lesions can show distinctive clinical presentations as in focal ischemia or haemorrhage. Contusional damage shows a certain predilection to frontotemporal lesions, typically causing impairments of attentional, executive, and memory functions, and potentially even more subtle deficits in social and moral behaviour [14,15]. On the other hand, DAI is more often associated with impaired consciousness in the acute phase [13] and—probably because of the interruption of large-scale intrinsic connectivity networks with specific interruptions of frontal and limbic connections—resulting in cognitive dysfunctions in the subacute and chronic phase, namely prominent executive and memory dysfunction [12,16,17,18]. 

However, the distinction between focal and diffuse lesions is somewhat arbitrary as they are often both present in the same patient, and are probably found in more than the 50% of TBI patients, demonstrated by a MRI study [13]. The cognitive disturbances after TBI are often accompanied by behavioural changes such as lack of initiative, irritability, and poor emotional control. Of note, compared with cognitive or behaviour problems and to outcome after stroke, persisting motor weakness after TBI has a relatively low incidence [19]. More often encountered in milder forms of TBI though, symptoms such as headache, dizziness, fatigue, sleep disturbances, and balance problems are common complaints and persist in a large number of patients in the long term [20,21]. Furthermore, psychological distress, depression, anxiety disorders, and substance abuse are highly prevalent with up to 75% of patients affected in one study [22].

In a typical case of severe TBI, the natural history of recovery consists of a period of impaired consciousness, sometimes accompanied by paroxysmal sympathetic hyperactivity (PSH), followed by a period of posttraumatic agitation (PA) and/or confusion with amnesia and, subsequently, by a period of post-confusional recovery of function, characterized by a diversity of cognitive, behavioural, emotional, and sensorimotor disturbances [11]. The medical aspects one is confronted with depend strongly on the individual course of recovery and the precise point in recovery at which rehabilitation is initiated. We will discuss some of these peculiarities of rehabilitation of TBI in more detail.

Furthermore, moderate-to-severe TBIs often co-occur with polytraumatic injuries including multi-organ injury, fractures, burns, or amputations. Therefore, the range of comorbid conditions and variations in the brain injury itself leads to a very individual array of physical, cognitive, behavioural, and psychosocial impairments in brain injured patients, which require a rehabilitation team specialized for neurorehabilitation of TBI.

## 3. Which Medical Aspects Are Special in the Rehabilitation of TBI?

### 3.1. Disorders of Consciousness (DOC)

Severe TBI can lead to prolonged periods of unconsciousness or coma. After a certain interval, post-traumatic coma—according to Plum and Posner, describing a state of «unarousable unresponsiveness» with eyes continuously closed and no clinical or electroencephalographical response to environmental or intrinsic stimulation [23]—often evolves to states with improved levels of arousal or consciousness: vegetative state (VS) or unresponsive wakefulness syndrome (UWS), and minimally conscious state (MCS), summarized as DOC.

VS indicates a state with no signs of conscious behavior, but patients show spontaneous eye opening, evidence of sleep–wake cycles in EEG, and typically do not require mechanical respiration—notably, no well-accepted definition of VS exists [24]. In 2010, a European task force on DOC introduced the term UWS in order to replace the often negatively connoted term of VS [25]. MCS is defined as “a condition of severely altered consciousness in which minimal but definite behavioural evidence of self or environmental awareness is demonstrated” and should be used only when clear and reproducible evidence of non-reflexive behaviour can be observed [26].

Clinical examination is still the mainstay of assessment and DOC evaluation. The Coma Recovery Scale-Revised (CRS-R) is often used in this context for assessment. It specifically focuses on and graduates signs of conscious awareness concerning motor and verbal behaviour, and thus allows the detection of changes in the clinical state [27]. Recently, more detailed scales have been developed and evaluated in DOC populations such as, for example, the DOC Scale (DOCS-25) and the Motor Behavior Scale (MBS) [28,29].

Assessed in a Norwegian population, three months after severe TBI, 2% of patients were in VS or MCS and less than 1% remained in a state of DOC after one year [30]. Both states can persist or can evolve into higher levels of consciousness, typically marked by the recovery to interact and communicate with the environment. The probability to emerge from these states and regain functional improvement typically correlates inversely with the duration of the DOC after TBI [31,32,33,34]—but not necessarily [35]. A large proportion of those who will show improvement do so in the first six months after injury [36]. Nevertheless, several well-documented cases have demonstrated that emergence from DOC can occur well after the first year after TBI [34,37,38,39]. In a study with very severe TBI patients, the authors found that patients with MCS or under anaesthesia three weeks after injury have a better prognosis than patients in UWS and coma [40]. The results are in accordance with the data of Katz and colleagues [32], who reported in 36 patients with DOC that the majority of their patients with MCS (72%) or confusional state/posttraumatic amnesia (CS/PTA, 58%) emerged from these states. Furthermore, the duration of MCS correlated with the speed of recovery of CS/PTA. Patients in VS for more than eight weeks never left CS/PTA level. In an older review, recapitulating data from 434 patients, emergence from VS had occurred in half of the patients, between 6 and 12 months in 6%, and beyond 12 months only in 1.6% [34]. 

Recently, data of a population of 110 patients with DOC due to TBI followed up for ten years was presented. More than half of the sample achieved near-maximal recovery by one year post-injury. In contrast, a subgroup of patients with delayed emergence from DOC (defined as command following later than four weeks after TBI) still showed significant functional improvement in the period between 2 and 10 years post-injury [33]. The number of patients in UWS/VS or MCS who will regain consciousness varies widely in the literature, probably because of the heterogeneity of definitions, the different time intervals at inclusion in the studies, and the lack of long-term follow-up in many studies. There may be also a selection bias in some studies (e.g., Katz’s population [32] was represented only by patients who were admitted to a specialized slow-to-recover brain injury program in an acute rehabilitation hospital). Finally, Hammond’s data [33] demonstrates the long period during which functional improvement is possible. Thus, individual outcome prediction is challenging.

Most treatment reports of DOC refer to single cases or small case series and only a few randomized controlled trials were reported, as reviewed comprehensively by Schnakers and Monti [41]. The best available data stems from an amantadine drug trial. In a randomized, multicentre study, 184 patients with VS or MCS received either amantadine (doses increased up to 200 mg twice a day) or a placebo for four weeks. Amantadine-treated patients with VS or MCS had shown earlier functional recovery on the Disability Rating Scale (DRS) as compared with patients under placebo [42]. 

A paradoxical effect of zolpidem with a temporary emergence of DOC had been observed in single cases. Subsequently, it has been investigated in two larger placebo-controlled trials [43,44]. Firstly, Whyte et al. found that in a cohort of 84 participants, at least four months after brain injury of different aetiologies, only four (5%) cases showed a clear response on the CRS-R indicating an improved level of consciousness, typically one to two hours after administration, but without prolonged effect [43]. Secondly, in a group of 60 patients with chronic DOC (31 had TBI as an underlying cause), the Liège group reported that only 12 patients (20%) showed improved behaviour and/or CRS-R, and only one patient had significant improvement in the form of functional object use [44]. Dopaminergic agonists (bromocriptine, levodopa/carbidopa, pramipexole) were studied only in small-sample studies, partially uncontrolled. They partially showed positive effects. However, sertraline, a selective serotonergic reuptake inhibitor, failed to improve the state of arousal in a series of 11 patients after severe TBI [45].

Multi-sensory stimulation programmes might be another promising approach, given their low potential to harm. In a preliminary report of a randomised placebo-controlled trial assessing the effect of familiar auditory sensory training, Pape and colleagues noted significant gains in the Coma-near-coma scale, representing arousal and awareness in patients with DOC [46]. Similar findings were reported by two groups using multimodal stimulation programmes [47,48]. The latter study showed that effects on the CRS-R, notably increased arousal and oromotor functions, were only found in MCS patients and not in VS patients [48]. 

Furthermore, it seems reasonable to provide care in a specialized institution given the particularities in this population. Besides the aforementioned pharmacological options and multi-sensory stimulation programmes [46,47,48], prevention and treatment of typical complications are often the main focus in these situations. It comprises means to maintain nutritional status and muscle mass, to prevent and treat spasticity and contractures, and to avoid circulatory deconditioning. The latter can be achieved by regular verticalisation, which, in one study, even had a positive effect on recovery on the CRS-R [49]—with no significant difference on the outcome between a conventional tilt table and one with an integrated stepping device, as shown in another study [50].

### 3.2. Paroxysmal Sympathetic Hyperactivity (PSH)

PSH can already occur in the acute phase of hospitalisation and may also be a problem in neurorehabilitation. PSH is defined as a recurrent and episodic syndrome, which includes hypertension, tachycardia, hyperthermia, diaphoresis, and hyperthermia [51]. Intermittent increased spasticity, dystonia, and extension or flexion posturing may also be observed. These symptoms may be an exaggerated response to some noxious external stimuli, such as sedation withdrawal, endotracheal tube suctioning, passive manipulation of the patient, urinary retention, constipation, or a loud acoustic environment. According to a systematic review of Perkes et al. [52], the majority of PSH cases are the result of TBI (79.4%), followed by hypoxic brain injury (9.7%) and stroke (5.4%). However, the lack of strict uniform diagnostic criteria in the different studies prevents comparison of PSH frequency. Therefore, even though there may be some consensus on signs and symptoms of PSH, the number, length, frequency, and severity of these have differed across studies.

### 3.3. Posttraumatic Agitation (PA)

PA is a subtype of delirium and may be a major problem in the management and rehabilitation of TBI. Patients show aggressive behaviour, disinhibition, or emotional lability. According to the literature, in the acute phases of brain injury, 35% to 96% of the cases may present PA. PA can persist during the recovery phase and the agitated behaviour profoundly affects the rehabilitation team. It should be mentioned that the diagnosis of PA is a “diagnosis of exclusion” after all medical (infection, metabolic and endocrine abnormalities, pain, and so on) and neurologic causes (e.g., hydrocephalus, intracranial lesion, migraine) have been ruled out [53]. It is generally recommended to reduce environmental stimuli by limiting noise and the number of visitors; furthermore, patients should be placed in a quite padded room, netbed, or vail bed. Patients may also need a one-to-one observation. Furthermore, it is helpful to remove noxious or potentially painful stimuli such as tubes, catheters, or restraints. The patient’s cognitive confusion may be reduced by maintaining consistency of the rehabilitation team and by a brief and clear communication style. Usually, pharmacological management of agitation and neurobehavioural disorders is needed. Numerous drugs have been tried in the management of aggression and several authors have developed guidelines for the treatment [54,55,56]. 

Luauté et al. [56] performed a systematic review of literature on the use of neuroleptics, antidepressants, beta-blockers, mood stabilizers, and other medications for irritability, aggressiveness, agitation, and other neurobehavioural disorders. They found 89 publications covering a total of 1306 people with TBI. They found insufficient evidence to standardize drug treatments for these disorders. Propranolol can improve aggression (evidence B grade). Carbamazepine and valproate seem effective for agitation and aggression and are recommended as first-line treatment (expert consensus opinion). They found no evidence for the efficacy of neuroleptics, which can be used in emergency situations. 

Finally, a Cochrane Review [57] showed that non-selective β-blockers (propranolol, pindolol) had the best efficacy for treatment of PA, while other drugs could not demonstrate firm evidence of their efficacy.

Treatment effects should be evaluated on a regular basis. Frequent clinical evaluation by the physicians is recommended. Easily applicable scales such as the Richmond Agitation Sedation Scale (RASS) [58] can be helpful for the nursing staff to assess the clinical course of agitation. It was originally validated to assess agitation and effects of sedative drugs in intensive care unit patients and is thus applicable in TBI patients too [59], but to our knowledge, has never been assessed systematically in neurorehabilitation. It consists of a 10-point scale, which allows description of patients’ behaviour from unarousable (−5) to combative (+4). A more detailed assessment is the Agitated Behavior Scale [60], which was specifically developed for use in TBI. It allows a more detailed assessment of agitation and typically requires an observation time of 10 min. It consists of 14 items describing different behaviours, and a rating from 1 to 4 is given depending on the presence of the described behaviour and its severity.

### 3.4. Posttraumatic Hydrocephalus (PTH)

PTH is the most common treatable complication during rehabilitation of TBI. The incidence of symptomatic PTH ranges from 0.7% to 29% [61]. Differences in diagnostic criteria and classification have contributed to the variation in reported incidence. The diagnosis of PTH is established using a combination of clinical, imaging, and physiological criteria.

PTH can be divided into two types: communicating and non-communicating PTH. In communicating hydrocephalus, the different parts of the ventricular system are interconnected and cerebrospinal fluid (CSF) flows from the ventricular system to the subarachnoid space. Communicating hydrocephalus is the most frequent type seen in TBI with blood products or fibrosis impeding the CSF flow into the bloodstream through the arachnoid granulations. This may also present as what is known as normal pressure hydrocephalus (NPH). In non-communicating or obstructive hydrocephalus, CSF flow is blocked from passing between the ventricles or exiting the ventricular system.

Risk factors for PTH include intracranial haemorrhage (particularly intraventricular haemorrhage), subarachnoid haemorrhage (SAH), meningitis, status after decompressive craniectomy, the duration of coma, and advanced age of the patient [62]. 

PTH may lead to clinical deterioration and poor outcome if untreated. A very early sign of developing PTH may be a stagnancy of improvement during early rehabilitation, discordant with injury severity. PTH may lead to clinical deterioration and poor outcome if untreated. Symptomatic PTH is to be distinguished from posttraumatic ventriculomegaly resulting from secondary atrophy. Symptomatic PTH patients are likely to improve when treated by shunting.

Kammersgaard et al., in 2013, prospectively followed 444 patients with severe TBI who required long-during rehabilitation [63]. They found that PTH occurred in 14.2% of the patients and 75% of the cases emerged during the rehabilitation. Patients who showed PTH were generally older, had more severe TBI, were more frequently in VS, and needed a longer rehabilitation stay. After adjusted analyses, however, older age and low level of consciousness were independently associated with PTH. This study shows that PTH is a complication occurring mainly during in-patient rehabilitation. The authors concluded that attention towards this complication should persist beyond the acute stage after TBI, particularly among older patients and patients with severely impaired consciousness.

Another cohort study with retrospective comparative analysis [64] found that in 701 patients admitted with TBI, 59 (8%) patients were diagnosed with PTH. Thirty-six (61%) patients with PTH emerged from PTA during rehabilitation. Finally, they found that earlier shunting predicted an improved outcome during rehabilitation. 

Tian and colleagues [65] reviewed the incidence of PTH in patients with traumatic SAH. They found PTH within three months of hospitalisation in approximately 12% of patients, with the majority occurring in two to four weeks after SAH. After a TBI, the latency of the appearance or detection of the PTH may be up to nine to twelve months [64,66].

Early signs and symptoms of non-communicating hydrocephalus include manifestations of increased intracranial pressure such as nausea, vomiting, lethargy, headaches, altered mental status, gait disturbances, and papilledema. 

As in the idiopathic form, secondary NPH typically presents with the clinical triad of gait ataxia, urinary incontinence, and dementia, with gait impairment the most likely to respond to surgical treatment. The cognitive abnormalities include poor activity initiation, psychomotor slowing, decreased attention, and forgetfulness. Ventriculoperitoneal shunts are used most commonly for posttraumatic NPH. Most patients who receive shunt placement will improve clinically and radiographically, as shown in a literature review [67]. However, even if neuroimaging shows a constellation compatible with NPH, the typical symptoms and signs of NPH can be concealed by the sequelae of TBI. Although it seems reasonable to consider shunt placement in this situation, there is no good evidence. In a retrospective series of 31 patients [68], 65% showed clear improvement—a less severe NPH according to neuroimaging and younger age seem to be predictive of better outcomes. 

### 3.5. Posttraumatic Neuroendocrine Disorders in TBI

Finally, during neurorehabilitation of TBI, one may be confronted with neuroendocrine disorders due to pituitary lesions. They are often missed and remain unrecognized. The pituitary gland seems particularly susceptible to acceleration–deceleration injuries due to the vulnerability of its vascular supply through the infundibulum and firm encasement within the sella turcica [69]. However, Klose and Feldt-Rasmussen are more sceptical and speculate that the frequent occurrence of anterior pituitary hormone alterations shortly after TBI seems quite similar to pituitary alterations in other critical diseases and can be considered to be a physiological adaptation to severe stress, as it often spontaneously remits [70]. The incidence of hypopituitarism is high in the acute TBI population, with a prevalence rate between 50% to 80% (for a review, see the work of [70]). For instance, Agha et al. [71] reported 18% incidence of growth hormone (GH) deficiency, 16% incidence of adrenocorticotropic hormone (ACTH) deficiency, 52% hyperprolactinemia, 40% with either diabetes insipidus (DI) or syndrome of inappropriate antidiuretic hormone (SIADH), as well as a notable reduction in serum thyroid-stimulating hormone (TSH).

In the chronic state of TBI, the literature is not uniform, and studies have been reported with prevalence rates of hypopituitarism from negligible to almost 70% [70]. The German Interdisciplinary Database contains a national registry of TBI and SAH patients, which was established in 2005. The Structured Data Assessment of Hypopituitarism after TBI and SAH resulted in a first report of 1242 patients [72]. The prevalence of hypopituitarism in the chronic phase (at least five months after the event) by laboratory values, physician diagnoses, and stimulation tests was 35%, 36%, and 70%, respectively. Furthermore, TBI patients with abnormal stimulation tests had suffered from more severe TBI than patients with normal stimulation tests. The authors concluded that hypopituitarism is a common complication of TBI and SAH. A follow-up publication [73] on patients with one to five or more years’ observation time showed the highest prevalence of neuroendocrine disorders one to two years post-injury, and it decreased over time only to show another peak in the long-term phase in patients with brain injury occurring ≥5 years prior to assessment. In the subgroup from one to two years after brain injury (n  =  126), gonadotropic insufficiency was the most common hormonal disturbance (19%) followed by somatotropic insufficiency (11.5%), corticotropic insufficiency (9.2%), and thyrotropic insufficiency (3.3%). In patients with observation time ≥5 years after brain injury, the prevalence of somatotropic insufficiency increased over time to 24.1%, whereas corticotropic and thyrotrophic insufficiency became less frequent (2.5% and 0%, respectively). The data show that neuroendocrine disturbances are frequent even years after TBI or SAH, in a cohort of patients who are still on medical treatment [73].

Clinical signs of hypopituitarism may be discrete, especially in a cohort of severely affected patients, and laboratory testing seems to be unreliable in the acute phase [70]. It is thus controversial whether laboratory screening or stimulation tests should be performed routinely. Klose and Feldt-Rasmussen concluded that available data do not support routine testing of GH, thyroid, and gonadal axes in the acute phase, but immediate management when adrenal and antidiuretic hormone insufficiency are clinically suspected [70]. Other authors highlight the need to screen for ACTH insufficiency in moderate to severe TBI [74], as decreased plasma cortisol levels were a predictor of mortality in another study [75]. They suggest repeated morning cortisol with a level <300 nmol/L being suggestive of adrenal insufficiency with the necessity for substitution [74]. Nevertheless, to our knowledge, a causal relationship between pituitary dysfunction and increased mortality has not been proven in larger studies.

## 4. Admission to Rehabilitation After TBI

### 4.1. Who Will Be Admitted to Neurorehabilitation?

Admission rates to neurorehabilitation for survivors of severe TBI differ widely between countries. Data from specialized trauma centres in Texas (U.S., 11 centres), Switzerland (12 centres), Norway (4 centres), and Denmark (4–5 centres) show rates of 45%, 44% (+16% non-specialized rehabilitation), 75% (+11% non-specialized), and 84%, respectively [76,77,78,79]. The comparability of these data is limited as a result of unequal populations (e.g., the Norwegian and Danish ones showing a notably lower mean age and a large proportion of younger patients, respectively), but these differences could also partly be explained by health system-inherent factors such as national guidelines, cost coverage/reimbursement, and centralization of institutions.

### 4.2. What Are the Admission Criteria?

A former review by Greenwald and Rigg [80] presented the following criteria for in-patient rehabilitation, presumably not based on evidence but derived from practical experience. (i) The patient has an acute disability that prevents him or her from returning home with family care; (ii) medical or surgical conditions are sufficiently stable to allow participation in therapies; (iii) the patient demonstrates the ability to participate in at least 1 h of therapy two times a day; (iv) the patient demonstrates the ability to make progress in acute care therapies; and (v) the patient has a social support system that will allow him or her to return home after reasonable improvement of function. 

Even more basically, the Canadian INESSS-ONF “Clinical Practice Guideline for the Rehabilitation of Adults with Moderate to Severe TBI” [81] defines that “Rehabilitation programs should have clearly stated admission criteria, which include a traumatic brain injury diagnosis, medical stability, the ability to improve through the rehabilitation process, the ability to learn and engage in rehabilitation and sufficient tolerance for therapy duration.”

### 4.3. Which Patients Should Be Excluded and Which Not—Despite Current Practice?

Older TBI patients are often denied access to specialized rehabilitation [82]. However, a comprehensive review of the literature from our group highlights that older TBI patients have the potential to make significant improvements over time, even though less favorable than younger patients [83].

From a practical perspective, reasons for exclusion from rehabilitation are premorbid states that either profoundly diminish the ability to participate in rehabilitation or that are associated with a poor long-term prognosis—for example, dependency of care before the TBI as in advanced dementia, or palliative situation due to advanced malignant tumours.

## 5. The Role of Timing and Rehabilitation Programmes on the Outcome in TBI Rehabilitation

There are currently no international guidelines regarding treatment in the early rehabilitation phase for patients with severe TBI. Only a few studies have investigated the effect of integrating rehabilitation into acute TBI care. 

A Cochrane Review evaluating multi-disciplinary rehabilitation for acquired brain injury in adults of the working age [84] found that intensive rehabilitation appears to lead to earlier gains. The efficiency of earlier intervention already in emergency and acute care has been supported by limited evidence. Furthermore, group-based rehabilitation in a therapeutic milieu (where patients undergo neuropsychological rehabilitation in a therapeutic environment with a peer group of individuals facing similar challenges) represents an effective approach for patients requiring neuropsychological rehabilitation following severe brain injury. The authors emphasized that not all questions in rehabilitation can be addressed by randomised controlled trials (RCTs) or other experimental approaches. Trial-based literature does not answer the question of which treatments work best for which patients over the long term, and which models of rehabilitation services represent the best value for money in the context of life-long care. 

In another review article [85], Turner-Stokes analysed the published evidence for the effectiveness of multidisciplinary rehabilitation following acquired brain injury in adults of working age. She used a synthesis of best evidence compiled from a Cochrane Review of RCTs and compared with literature assembled for the U.K. National Service Framework for long-term neurological conditions, using a typology based on evaluation of research quality irrespective of study design.

She emphasized that the rehabilitation of TBI is a complex process with several major challenges for clinical research that tend to confound traditional RCT designs. Three characteristics of rehabilitation studies make it difficult use only the RCTs approach because it cannot be applied to address all the questions that need to be answered in TBI rehabilitation [85]; these include the following: (i) The numbers of studies are comparatively small, and there is marked heterogeneity with respect to the severity of TBI, the intervention and the clinical setting. Furthermore, the outcomes may be different at each stage of recovery. (ii) There are ethical considerations, as many patients with moderate to severe TBI may lack the cognitive capacity to give fully informed consent to participate in research. Furthermore, the increasing evidence for effectiveness of multi-disciplinary rehabilitation in many conditions (particularly stroke) makes it unethical to randomize patients to “no treatment” or even “standard” care. (iii) The timescale over which rehabilitation may have its effects (months or years) is usually much longer than any funded research project. They analysed both RCT and non-RCT-based research to support the effectiveness of rehabilitation for adults with TBI using the GRADE system proposed by the GRADE Working Group (Grading of Recommendations Assessment, Development, and Evaluation) [86]. The recommendations for clinical practice are presented in Table 1.

They found high evidence and, therefore, a strong recommendation for more intensive rehabilitation programmes in severe TBI, which are associated with faster functional gains. Moderate to high evidence exists for specialised or vocational rehabilitation programmes, whereas moderate evidence exists for programmes that continued outpatient therapy and for early rehabilitation programmes. 

The concept of very early rehabilitation is supported by more recent studies, for example, by Andelic et al. [87]. They investigated, in a cohort of 61 surviving patients with severe TBI, whether a continuous chain of rehabilitation starting in the acute phase improves the functional outcome of these patients, compared with a broken chain of rehabilitation that starts in the subacute phase after TBI—31 patients were in the early rehabilitation group and 30 patients were in the delayed rehabilitation group. The outcome was assessed 12 months after TBI using the GOSE and DRS. A significant favourable outcome (GOSE 6–8) occurred in 71% of the patients of the early rehabilitation group versus 37% in the delayed rehabilitation group. The same was true for the DRS score, which was significantly better in the early rehabilitation group. A recent review and meta-analysis in this field concluded that the available evidence indicates that early onset neurorehabilitation in a trauma centre and more intensive neurorehabilitation in the post-acute setting promote functional recovery [88].

Rehabilitation institutions are frequently located elsewhere than the acute care institutions and lack the possibility of intensive monitoring of patients with early medical complications (e.g., PSH, CS/PTA, PA). Therefore, in these conventional settings, the time lapse between the occurrence of brain injury and admission seems to depend primarily on the severity of brain injury and intercurrent complications [89].

## 6. Intensity and Duration of Interprofessional In-Patient Rehabilitation

### 6.1. Intensity

The question of optimal intensity of therapies in neurological rehabilitation is difficult to answer, given the heterogeneity of patients. Yet, several studies found evidence of earlier gains in more intensive therapy in TBI. First, Zhu et al. [90] compared more intensive (4 h/day) to conventional (2 h/d) rehabilitation in 68 patients. A significantly greater number of patients with a more intensive programme reached maximal Functional Independent Measure (FIM) and Glasgow Outcome Scale (GOS) scores within three months with no significant effects at later points in time. Shiel and colleagues [91] investigated enhanced intensity versus “routine” multi-disciplinary rehabilitation in two study centres in a total of 51 patients. They found significantly greater improvement in functional scores (FIM + FAM) in the enhanced intensity group compared with the control group (absolute or relative quantities of therapy not specified).

In contrast, a recent study by Hart and colleagues [92] compared outcomes one year after severe TBI in 274 patients between two different study site—one in the U.S. and one in Denmark. Even though the Danish site provided significantly greater intensity and duration of rehabilitation, there were no site differences in either functional or emotional outcome at 12 months; as the Danish population was more severely affected, adjustment for patient/injury characteristics was performed. It is difficult to draw conclusions from this study, but pure intensity- and/or duration-related improvements could not have been shown.

Nevertheless, in our practical experience with patients in the first few weeks after severe TBI, they can show limited ability to participate in longer therapy sessions and in group therapies with significant fatigability. This can be an important limiting factor for higher therapy intensities.

### 6.2. Duration

On the one hand, more intensive therapy potentially can reduce the LOS. Slade et al. compared two groups with different intensities of multi-disciplinary rehabilitation, with one benefitting from 30% more therapy time than the other [93]. After statistical correction for confounding factors (impairment mix, community delays, missed treatment) with a multiple regression model, a 14-day reduction of the LOS for the group with more intensive treatment with comparable functional scores (Barthel index) could be demonstrated. Similarly, in the abovementioned data from Shiel [91], LOS was significantly reduced in one group with more intensive treatment, but only in one study site. In contrast, in the other study center, LOS was even longer, which the authors explained with a disproportionate number of very severely affected patients.

On the other hand, as reported based on observational data [94,95], longer rehabilitation stays can be associated with more important improvements as measured with DRS or FIM, respectively. Correspondingly, Foy and Somers could demonstrate an association between LOS and functional improvements in young adults participating in an in-patient programme with 5 h of therapy and/or education daily [96]. Nevertheless, this cannot be translated into simple LOS-related improvements; hypothetically, it could also imply that patients with less important progress during rehabilitation were dismissed earlier in those studies. However, Turner-Stokes could calculate that rehabilitation in-patient treatment lasting longer than four months for patients with complex neurological disabilities (but only 18% TBI in the presented sample) can be cost-effective, as it does effectuate reductions of dependency and subsequently the cost of long-term care in selected patients [97].

In clinical settings, different factors influence the LOS in in-patient rehabilitation: (i) health system-related factors such as, for example, predefined maximum LOS, limited capacity of the rehabilitation facilities, availability of intensive outpatient/community programmes and availability of nursing/care home places, respectively; (ii) patient-related factors such as, for example, individual goals and motivation, the availability of support by families and caregivers, and occupational status and position, respectively.

## 7. Are There Any Specific Rehabilitation Approaches Superior to Another?

Systematic reviews by the Institute of Medicine [98] and by Brasure et al. [99] stated that the existing, rather limited evidence does not allow conclusions about comparative effectiveness of different rehabilitation programmes, for example, in terms of activities and participation as outcome measures. These findings have to be put into perspective with the above-mentioned difficulties concerning rehabilitation trials. 

For instance, Cicerone and colleagues could show that an intensive cognitive rehabilitation program as compared with standard rehabilitation may lead to earlier patient productivity [100]. In addition, advances during rehabilitation are not always necessarily measurable by the means of scales, but can be of significant value for the individual. In their review on evidence-based cognitive rehabilitation, Cicerone et al. concluded in 2011 that there was substantial evidence to support interventions for attention, memory, social communication skills, executive function, and comprehensive-holistic neuropsychological rehabilitation after TBI [101]. In accordance with this, the guidelines by an international group of researchers and clinicians (INCOG) recommends multidisciplinary cognitive rehabilitation tailored to patients’ neuropsychological profiles, premorbid cognitive characteristics, and goals for life activities and participation [102]. Similarly, the Scottish guideline on brain injury rehabilitation in adults demands that these interventions have to be embedded into comprehensive/holistic neuropsychological rehabilitation programmes, involving an interdisciplinary team using a goal-focused programme that has the capacity to address cognitive, emotional, and behavioural difficulties with the aim of improving functioning in meaningful everyday activities [103].

This neurorehabilitation team typically consists of a specialized nursing team, physiotherapists, occupational and speech therapists, neuropsychologists, and physicians; optionally, it could include nutrionists, social workers, and recreational/vocation therapists. The team regularly unites to reassess the patient’s state and progress and to discuss and redefine treatment goals. Frequent education of the patient and his family is suggested in order to foster comprehension of and compliance to the treatment plan and possible outcomes. 

## 8. Conclusions

Neurologists and other physicians involved in TBI rehabilitation should be aware of the particularities concerning lesion patterns, clinical presentations, and the typical sequence of recovery in moderate to severe TBI, as well as the above-mentioned frequent complications that may interfere with rehabilitation progress. Considering the heterogeneity of physical, cognitive, behavioural, and psychosocial sequelae after TBI, it is in the experience of the authors, and represented in several guidelines too [81,102,103], that rehabilitation has to have an individualized and focused approach on the patients’ aims, needs, resources, and deficits—in accordance with the International Classification of Functioning, Disability, and Health (ICF). Furthermore, early rehabilitation can improve patients’ outcomes.

## Figures and Tables

**Table 1:** Recommendations for clinical practice (using the GRADE system) for different neurorehabilitation approaches in TBI. TBI—traumatic brain injury; GRADE—Grading of Recommendations Assessment, Development, and Evaluation.

Quality of Evidence	Rehabilitation	Patient Category	Outcome	Potential of Cost Savings	Recommendation (GRADE System)
**High**	Intensive	Severe TBI	Earlier gain in independence Reduced length of stay (LOS) in hospital	+	Strongly recommended
**Moderate/high**	Specialist	Very severe/severe TBI	Improved independence Reduced ongoing care	++	Recommended
	Specialist vocational programmes	Moderate/severe TBI	Gain in productivity	++	Strongly recommended
**Moderate**	Early	Severe TBI	Earlier gain in independence Reduced LOS in hospital	+	Recommended
	Community based	Moderate/severe TBI	Improved productivity	++	Recommended
**Low/moderate**	Behavioural management programmes	TBI with severe behavioural problems	Improved social behaviour reduced ongoing care support	+	Recommended
	Late and ongoing rehabilitation	Moderate/severe TBI with enduring disability	Maintenance of independence/productivity	+/−	Conditionally recommended
